# Draft Genome Sequences of 15 *Staphylococcus aureus* Isolates Recovered from Raw Milk and Associated Milk Filters from Victoria, Australia

**DOI:** 10.1128/genomeA.01463-16

**Published:** 2017-01-12

**Authors:** Kate McMillan, Theodore R. Allnutt, Edward M. Fox

**Affiliations:** a CSIRO Agriculture and Food, Archerfield BC, Queensland, Australia; b CSIRO Agriculture and Food, Werribee, Victoria, Australia; c School of Medicine, Deakin University, Waurn Ponds, Victoria, Australia

## Abstract

This study describes draft whole genomes of 15 *Staphylococcus aureus* isolates from dairy farms located in Victoria, Australia. Two novel sequence types (ST3183 and ST3184) were identified among these isolates.

## GENOME ANNOUNCEMENT

*Staphylococcus aureus* is a Gram-positive bacterium that can cause mastitis in dairy cattle and food poisoning in humans. Draft genome sequences were generated for 15 *S. aureus* isolates recovered from raw milk or their associated milk filters from dairy farms in Victoria (1). This included eight bovine farm isolates, five caprine farm isolates, and two ovine farm isolates.

Genomic DNA extracts were prepared using the DNeasy blood and tissue kit (Qiagen, Hilden, Germany) and sent to the Ramaciotti Centre for Genomics (University of New South Wales, Sydney, Australia) for library preparation (Nextera XT library prep kit, Illumina, San Diego, CA, USA) and whole-genome sequencing on the Illumina MiSeq platform. Quality filtering and adapter trimming was done on Trimmomatic version 0.22 software. The preprocessed reads were then *de novo* assembled using the SPAdes version 2.5.1 genome assembler tool to produce contigs in FASTA format. The 15 genome assemblies generated varied in size from 2.70 Mb to 2.81 Mb. The number of contigs for each genome ranged from 49 to 220, with an average coverage of >20× and a maximum coverage of 87×. The contig *N*_50_ ranged from 31,103 bp to 294,781 bp with an average GC content of 32.75%. Assembly details are shown in Table 1. Automatic genome annotation was conducted on the FASTA files using RAST, and GenBank files were generated.

**Table 1:** NCBI accession numbers and quality metrics of 15 *S. aureus* isolates

Isolate	NCBI BioSample no.	GenBank accession no.	Genome size (bp)	G+C content (%)	No. of contigs	*N*_50_ (bp)	Median coverage (×)
Sa12-001	SAMN05188390	MAQG00000000	2,704,018	32.7	49	294,781	42
Sa12-002	SAMN05188391	MAQH00000000	2,697,729	32.8	100	175,785	87
Sa13-001	SAMN05188392	MAQI00000000	2,711,181	32.7	127	62,005	30
Sa13-002	SAMN05188393	MAQJ00000000	2,714,960	32.8	220	31,103	20
Sa13-003	SAMN05188394	MAQK00000000	2,805,285	32.9	118	107,158	43
Sa13-004	SAMN05188395	MAQL00000000	2,801,626	32.8	124	105,140	50
Sa13-005	SAMN05188396	MAQM00000000	2,800,437	32.8	123	122,121	60
Sa13-006	SAMN05188397	MAQN00000000	2,771,834	32.8	94	83,898	37
Sa14-001	SAMN05188398	MAQO00000000	2,772,098	32.8	62	130,009	44
Sa14-002	SAMN05188399	MAQP00000000	2,719,143	32.7	62	192,514	52
Sa14-003	SAMN05188400	MAQQ00000000	2,745,709	32.7	61	202,483	46
Sa14-004	SAMN05188401	MAQR00000000	2,718,606	32.7	213	31,291	20
Sa14-005	SAMN05188402	MAQS00000000	2,774,779	32.8	81	194,021	56
Sa14-006	SAMN05188403	MAQT00000000	2,800,711	32.8	87	240,117	61
Sa14-007	SAMN05188404	MAQU00000000	2,800,727	32.8	85	240,117	86

Multilocus sequence types were assigned according to the *S. aureus* multilocus sequence type database (http://saureus.mlst.net) (2). Seven sequence types (STs) were identified, including two novel STs (ST3183 and ST3184) (3). All ovine and caprine isolates were ST133 or a highly related variant (ST3184), whereas bovine isolate STs were diverse (ST1, ST5, ST8, ST705, and ST3183). A single isolate (MAQP00000000, Sa14-002) harbored the *blaZ* gene, which confers penicillin resistance; penicillin resistance was also confirmed phenotypically. Two isolates, MAQQ00000000 (sa14-003) and MAQR00000000 (sa14-004), harbored the enterotoxin gene cluster (*egc*) variant 1, which carries *seg* to *sei* along with *yent1* and *yent2*. A diverse range of staphylococcal enterotoxins were found among bovine isolates, which included *sec-sed*, *seg*-*sej*, *sel*-*seo*, and *ser*; this was in contrast to caprine and ovine samples, which contained only *sec* and *sel*. A high proportion of caprine and ovine isolates contained TSST-1 (*n* = 4), relative to bovine isolates (*n* = 1).

### Accession number(s).

This whole-genome shotgun project has been deposited at DDBJ/ENA/GenBank under the accession numbers listed in Table 1. The versions described in this paper are the first versions.
